# Anomalous Twin Pregnancy

**Published:** 1879-03

**Authors:** 


Anomalous Twin Pregnancy.—The following case being of some rarity in its details and occurrence, I venture to submit it, thinking it might interest those more especially engaged in obstetric practice.

In twin pregnancy, the development of the children does not always advance *pari passu*, as the following case will illustrate. On October 18th, I attended a Mrs. P., aged 26, in her first parturition, the labor being tedious, and the head remaining a long time in the cavity of the pelvis. There being inertia from exhaustion, I applied Ziegler's forceps and delivered without laceration or difficulty. Five minutes afterwards, I removed the placenta by traction on the funis. There was no hemorrhage of importance. The following day the patient seemed very comfortable, with the exception of a sensation of weight in the region of the bladder, whereupon I introduced a female catheter and drew off a pint and a quarter of urine. On the 30th the patient complained of nothing, and felt tolerably well; so likewise on the morning of the 31st. During the afternoon of this day, I was sent for to see a shrivelled, wasted fœtus, with funis and placenta all complete, which was expelled four days after the extraction of the full-grown, living, male, healthy child. The hæmorrhage was but slight, and there was scarcely any pain.

This fœtus was evidently a twin, conceived, no doubt, at the time of its brother, and had, it appeared, developed normally up to about six months, when it ceased to grow, and became gradually flatly compressed and shrivelled, but not putrid, having at the time of expulsion a faint odor, and being of a light flesh color.

The causes adduced for the death of one fœtus are, in the main, four: 1. One child appropriates to itself all the nourishment, at the expense and destruction of the other; 2. Pressure of the uterus and one fœtus on the other; 3. Disease originating in the fœtus or placenta; 4. Separation of placenta (accidental) from the uterine wall. In this case there would be, as a rule, hemorrhage, slight or excessive, during pregnancy.

The case in point seems to me to be referable to the second cause, viz., that of pressure; for there was no history of syphilis—no history of hemorrhage during pregnancy, or of a fall or concussion of any kind. On the contrary, the patient had especially good health during pregnancy; no old clots were met with at parturition. The placenta of the dead child was somewhat tough and pale, shrivelled, entire; there was no trace of lesion about it. It remained adherent to the uterus until the time of expulsion *per vaginam*, which was accompanied with slight hemorrhage. The patient had no subsequent troublesome symptoms, involution proceeding normally.

